# Astrocyte Activation Persists after Recovery of Myelin and Motor Deficits in the Cuprizone Model: a Longitudinal PET and CNS Tissue Analysis Study

**DOI:** 10.1007/s11307-026-02098-5

**Published:** 2026-03-24

**Authors:** V. Pegoretti, A. S. Boerema, J. W. A. Sijbesma, L. E. R. Dietz, N. Alberts, W. Douwenga, U. L. M. Eisel, E. F. J. de Vries

**Affiliations:** 1 Department of Molecular Neurobiology, Groningen Institute of Evolutionary Life Science (GELIFES), University of Groningen, Groningen, The Netherlands; 2 Department of Nuclear Medicine and Molecular Imaging, University Medical Center Groningen, University of Groningen, Groningen, The Netherlands; 3 Applied Research Center, Van Hall Larenstein University of Applied Science, Leeuwarden, The Netherlands

**Keywords:** [^11^C]MeDAS, [^11^C]PK11195, Balance beam test, Cuprizone, Demyelination, MOSS test, Neuroinflammation, Positron emission tomography, Remyelination

## Abstract

**Purpose:**

The cuprizone (CPZ) mouse model for multiple sclerosis enables researchers to investigate the underlying mechanisms of demyelination and remyelination, as well as the effect of therapeutic interventions thereon. Over the last five decades, research revealed detailed analyses of brain tissue derived from CPZ-fed animals at different times of de- and re-myelination. Yet, longitudinal analysis of the effects of a CPZ diet on locomotor performance, myelination and neuroinflammation over a two-month recovery period are still unexplored.

**Procedures:**

In this study, we comprehensively examined behavioural and neuropathological changes in the CPZ mouse model for a follow-up period of two months. We combined a longitudinal PET imaging approach with a more traditional approach of obtaining tissue and performing immunohistochemistry and behavioural tests in cohorts.

**Results:**

We found that mice fed with 0.2% CPZ for 5 weeks showed transient motor deficits which recovered quickly after cuprizone was removed from the diet. Similarly, remyelination also promptly restored myelin content after CPZ toxic insult, as seen by PET imaging with [^11^C]MeDAS and myelin histological staining. Remarkably, five weeks of CPZ feeding led to persistent glial activation, especially in the corpus callosum, for at least two months. This effect was measured both with [^11^C]PK11195 PET and with immunohistochemical staining for microglia and astrocytes.

**Conclusions:**

Taken together, this longitudinal study reveals that a five-week CPZ diet leads to transient motor and loss of myelin, which recover quickly after CPZ is removed. In contrast, neuroinflammation persists for at least two months following exposure and is mainly driven by astrocyte activation. The study warrants deeper analysis of the sustained neuroinflammatory response in the context of demyelinating diseases.

**Supplementary Information:**

The online version contains supplementary material available at 10.1007/s11307-026-02098-5.

## Introduction

Primary demyelination consists in the destruction of myelin and is characteristic of different demyelinating diseases, such as multiple sclerosis (MS), neuromyelitis optica spectrum disorders (NMOSD) and acute disseminated encephalomyelitis (AEM) [1]. Even though some of the pathological hallmarks may be common to different diseases, demyelinating events can have distinct aetiologies and be driven by various pathological mechanisms and genetic defects [2, 3]. Demyelination is characterized by the death of oligodendrocytes and the destruction of myelin layers, causing white matter (WM) lesions and eventually impaired signal conduction. In certain clinical conditions, demyelination is a reversible process where spontaneous regeneration of myelin sheaths at the lesion site occurs. Oligodendrocyte precursor cells (OPC) and neural stem cells (NSC) migrate to the demyelinated area and differentiate into mature, myelin-forming cells able to remyelinate the naked axons [4]. Oligodendrocytes surviving demyelination are also capable of forming new myelin sheaths but less efficiently [5].

In MS, remyelination failure dictates the degree and irreversibility of axonal damage and neurodegeneration which in turn are responsible for progression of the disease [6, 7]. It is therefore essential to understand remyelination processes to prevent the drastic consequences of demyelination. For this purpose, several *in vivo* models, such as the cuprizone (CPZ) mouse model, were developed [8, 9]. Similar to demyelinating diseases in humans, CPZ-fed animals show anxiety-like behaviour, poor motor coordination and working memory function [10, 11]. CPZ is a copper chelator which induces iron-mediated lipid peroxidation and loss of oligodendrocytes via ferroptosis-mediated cell death [12]. CPZ diet causes mitochondrial impairments [13], oxidative damage to oligodendrocytes [14] and inflammation, which stimulates glial proliferation, and clearing of myelin and cellular debris [15]. An interesting feature of the CPZ model is its reversibility. Even though feeding animals for several weeks with CPZ induces extensive demyelination and gliosis, remyelination spontaneously starts already around the fourth week of CPZ feeding [16]. Previous studies have provided detailed analyses of brain tissue from CPZ-fed animals at various stages of de- and remyelination [9, 15]. However, the long-term effects after a five-week CPZ diet have remained relatively unexplored. Other studies show that axonal degeneration and glial activation are still present seven months after a five-week CPZ diet, even though myelin density appears normal [17]. Locomotor [18] and cognitive [19] performance are also impaired six weeks after CPZ intoxication. These studies emphasize the need for a detailed analysis of the neuropathological and behavioural changes over time triggered by CPZ-induced demyelination.

To longitudinally assess the extent of demyelination, disease progression and efficacy of treatments in MS patients, structural magnetic resonance imaging (MRI) is regularly used in healthcare while positron emission tomography (PET) is still mainly employed in clinical trials [20]. MRI has superior anatomic resolution with good soft tissue contrast when compared to PET. Although the latest technological advances increased the sensitivity of MRI techniques in detecting disease activity [21], the molar sensitivity of PET for imaging of metabolites and molecular probes is still orders of magnitude higher than that of MRI [22]. PET imaging with its many available radioligands, aiming for imaging of several aspects of neurological diseases, can be a valuable tool for diagnosing and analysing disease development. Several PET tracers were developed to image myelination or CNS inflammation and tested in both pre-clinical and clinical studies [23, 24]. For instance, [^11^C]MeDAS is a PET tracer that binds to the β-sheet structures found in myelin basic protein (MBP). Previous studies showed that [^11^C]MeDAS can be used to detect myelin density in the CPZ model [25] but also in MS patients [23]. Moreover, imaging neuroinflammation can also improve diagnosis and assessment of disease progression [24]. Overexpressed mainly by reactive glial cells [26, 27], the translocator protein (TSPO) on the outer mitochondrial membrane has been a long-standing target for PET ligands [28]. For instance, the PET tracer [^11^C]PK11195 binds to TSPO and thus can help to track the inflammatory response over time in preclinical [29] and clinical studies [30]. Nevertheless, [^11^C]MeDAS and [^11^C]PK11195 PET tracers have not been used to measure myelination and neuroinflammation over a long time period in preclinical models of demyelinating diseases.

This study aimed to examine behavioural and neuropathological changes before, during and up to two months after the end of CPZ feeding to reveal long-term changes in myelination, neuroinflammation and physical motor performance. By using both PET imaging and histology, we found that loss of myelin recovered substantially faster than glial activation in this model. Furthermore, CPZ-induced motor deficits measured with MOSS and balance beam tests are reversible, with balance beam being a more sensitive test to discriminate between remyelination phases. These findings further expand our understanding of long-term changes after CPZ feeding and the imaging techniques used to measure these.

## Methods

### Experimental Design and Cuprizone Feeding

**Fig. 1:**
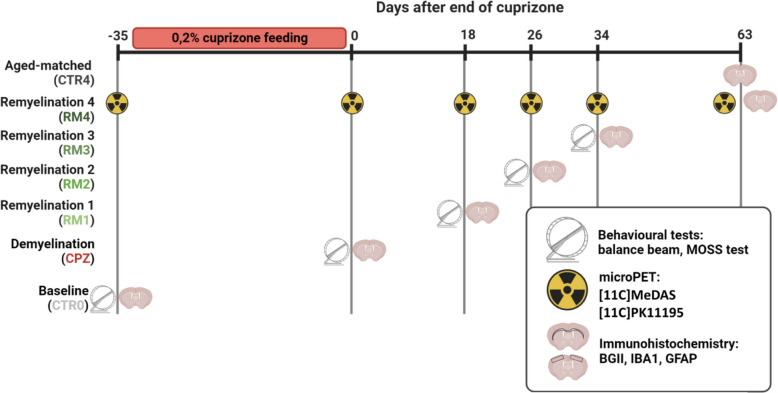
Timeline and design of the experiment. C57BL/6 male mice were fed with 0.2% cuprizone for five weeks and sacrificed before (CTR0, grey) or at the end of cuprizone feeding (CPZ, red) as well as 18 (RM1, green), 26 (RM2, green), 34 (RM3, green) and 63 days (RM4, green) after end of cuprizone feeding. PET imaging with [^11^C]MeDAS and [^11^C]PK11195 was performed longitudinally only in animals belonging to the RM4 group. Behavioural experiments were performed before sacrifice, after which brains were collected and stained for relevant markers analysed in the corpus callosum and/or cortex areas (see legend)

Male C57BL6/J mice were bought from Jackson Laboratories and included in the study at the age of 5 weeks. Mice were single-housed at constant temperature (20 ± 1 °C at a 12-h light/12-h dark cycle. Food and water were available ad libitum throughout the experiment. At 8 weeks of age, mice were fed with 0.2% cuprizone (Sigma-Aldrich) mixed in standard chow powder for five weeks (CPZ group) to induce global demyelination. A fresh stock cuprizone-supplemented powder food was prepared weekly and stored in a light- and airtight container at 6 ºC. Powder food in the mouse cages was replaced daily. After CPZ feeding, mice were fed with standard chow powder and sacrificed at different time points of remyelination (RM): 18 days (RM1 group; n = 10), 26 days (RM2 group; n = 10), 34 days (RM3 group; n = 10) and 63 days (RM4 group; n = 8) after the cessation of cuprizone feeding (Fig. 1). Control mice were not exposed to cuprizone and were sacrificed either at 8 weeks of age, just before the start of CPZ feeding in the RM groups (CTR0 group; n = 10) or at the same age as the RM4 group (CTR4 group; n = 6). The end of the cuprizone diet (CPZ group) and the first remyelination timepoint (RM1) were selected based on previous literature on the well-studied cuprizone model [8, 9, 11, 15]. In the absence of prior studies guiding optimal time-point selection, remyelination-phase time points (RM2–RM4) were chosen to provide detailed coverage of the first month, with repeated assessments at 8-day intervals. The final time point was selected after an additional month (RM4) to cover a total remyelination period of two months. Before sacrifice, behavioural tests were carried out. As shown in Fig. 1, mice belonging to the RM4 group (n = 8) were subjected to longitudinal PET imaging at different experimental time points and sacrificed 63 days after cuprizone feeding. These mice and the age-matched control group (CTR4) were not exposed to behavioural tests because of ethical reasons concerning the cumulative discomfort of the combination of behavioural test with longitudinal PET imaging. The general appearance and body weight of each animal was recorded daily. All aspects of animal care and treatment adhered to national law and ethical guidelines. Animal experiments were approved by the institutional animal care and use committee of the University of Groningen (license number DEC-5902i). Experimental procedures and protocols were discussed and optimized with the Animal Welfare Body of the Central Animal facility of the University Medical Center Groningen.

### Behavioural Tests

#### Balance Beam Test

The balance beam test is a test focusing on hindlimb function [31] and can also be used to measure the effects of cuprizone feeding on motor performance in mice [32]. The test was performed according to the protocol described previously [33, 34]. A 100 cm long, 0.5 cm wide metal beam was elevated 60 cm above the floor. At one side of the beam a platform with the home cage was located, incentivizing mice to cross the beam and reach their cage. On the other side of the beam a translucent plastic screen was placed that prevented mice from leaving the beam. A camera was placed behind the screen to film each trial. Mice were first trained to traverse by placing them on the beam at increasing distances from the home cage. Subsequently, three complete trials at full distance were measured for the mice included in the behavioural analysis. (Fig. 1). The trials were filmed and an observer blinded for the experimental group scored the trials in random order. Time to cross the beam and missed steps were registered. The average of the three trials was used in the analysis.

#### Motor Skill Sequence (MOSS) Test

The MOSS test was carried out as previously described [35, 36]. Mice were first habituated for two weeks to normal running wheels in their cage. Wheels were then replaced for one week with more intricate wheels with irregularly placed bars requiring higher-level interhemispheric paw coordination, amplifying potential motor impairments caused by interhemispheric white matter lesions in the corpus callosum. Wheel revolutions were registered by reed switches in the cage registering a small magnet in the running wheel. Revolutions were registered in 2 min bins and summed per day.

#### [^11^C]PK11195 and [^11^C]MEDAS Positron Emission Tomography

Microglia activation was longitudinally monitored with [^11^C]PK11195 PET, as described previously [37]. CNS myelin content was measured with [^11^C]MEDAS PET, as described previously [25]. Longitudinal PET imaging at six experimental time points was performed only on mice belonging to the RM4 group (n = 8, Fig. 1). These animals were subjected to PET scans with both tracers, allowing sufficient time between PET scans for radioactivity decay (max 32 h). In brief, mice were anesthetized with isoflurane (induction 5%, maintenance 1.5%) in medical air (60% O_2_-enriched) and injected with 6.92 MBq (SD 5.27 MBq) [^11^C]PK11195 or 3.40 MBq (SD 2.49 MBq) [^11^C]MEDAS (decay corrected to the start of the PET acquisition) via penile vein injection. Four mice per scan were placed, in prone position, on heating pads, in the dedicated small animal PET camera (MicroPET Focus 220, Siemens Medical Solutions, Malvern, Pennsylvania). For [^11^C]PK11195, an emission scan of 20 min was started 30 min post tracer injection. For [^11^C]MEDAS, an emission scan of 30 min was started 25 min post tracer injection. After completion of the emission scan, a 10 min transmission scan was obtained for attenuation and scatter correction, using a cobalt-57 point source. Positron emission tomography scans were iteratively reconstructed (OSEM2D, 4 iterations, and 16 subsets) into 10 min frames after being normalized and corrected for attenuation and decay of radioactivity. The emission data 30–40 min ([^11^C]PK11195) and 45–55 min ([^11^C]MEDAS) post-injection was used for analysis. Images with a 512 × 512 × 95 matrix, a pixel width of 0.475 mm, and a slice thickness of 0.796 mm were obtained. Individual animal head regions, containing the brain, were cropped from the image using AMIDE 1.05 Software [38]. The images were resliced into cubic voxels (0.2 mm) and converted into standardized uptake value (SUV), defined as: [tissue activity concentration (MBq/cm^3^)]/[(injected dose (MBq)/body weight (g)]. Brain images were analysed as described previously [34]. For each tracer, one image was randomly selected and all other individual images of that tracer were automatically co-registered to this image using VINCI 4.39 software (Max Planck Institute for Metabolism Research, Germany). Then, a tracer-specific average image of the co-registered individual images was created. The average image of each tracer was manually aligned to the C57Bl6/J magnetic resonance imaging (MRI) brain atlas previously published by Ma et al [39]. The original individual PET images were then co-registered to the average image of the same tracer, aligned with the MRI atlas. Atlas [39]-based regions of interest (ROI) were used to extract the SUV values for different brain regions from all scans. Average SUV values for all ROI’s are reported in Tables S1 and S2. To obtain a value for the whole brain, the regions were summed, excluding the olfactory bulb and the caudal brainstem, as these regions are most affected by partial-volume effects in mice.

### Histology, Immunohistochemistry and Image Analysis

#### Tissue Processing

After administering a lethal dose of pentobarbital intraperitoneally at the appropriate experimental time points (Fig. 1), animals underwent transcardial perfusion to systemically deliver first a solution of heparinized saline, followed by 4% paraformaldehyde (PFA) in PBS. Subsequently, the brains were removed, post-fixed for 24 h in 4% PFA in PBS, subjected to multiple washes in PBS, and then incubated overnight in a 30% sucrose solution to enable cryopreservation. Following additional washes with double distilled water, the brains were frozen in liquid nitrogen and stored at −80 °C until further use.

#### Immunohistochemistry

Coronal brain Sects. (25 µm thick) were cut using a cryostat and stored free-floating in PBS at 4 °C. To block unspecific binding and enhance permeability, sections were pre-incubated with 10% normal donkey serum and 0.5% Triton-X100 in PBS for 1 h. Subsequently, the sections were incubated at 4 °C with IBA1 (rabbit anti-mouse IBA1, Wako Chemicals, 1:2.500, catalogue nr: 019–19741) and GFAP (mouse anti-mouse GFAP, Sigma-Aldrich, 1:10.000, catalogue nr: G3893) antibodies diluted in the blocking solution for 72 h or overnight, respectively. Three washing steps preceded the incubation with the secondary antibodies (goat anti-rabbit IgG or goat anti-mouse IgG biotin-conjugated, Jackson ImmunoResearch, 1:500) for 2 h at room temperature. After three washing steps, sections were incubated with avidin–biotin complex (ABC, 1:500, Vector Laboratories) for 1 h at room temperature followed by three washing steps and subsequent incubation with 3,3'-diaminobenzidine-tetrahydrochloride (DAB, 0.7 mg/ml, Sigma-Aldrich) to generate a brown precipitate. Each washing step was performed with 0.5% Triton-X100 in PBS for 5 min. Mosaic images of whole brain sections at 100 × magnification were captured with a Leica DMI6000B fluorescence microscope equipped with a monochrome digital camera (Leica Microsystems). The overall signal coverage of IBA1 or GFAP staining in the corpus callosum (CC) or cortex was quantified using ImageJ. IBA1-positive cells in regions of interest in the CC were manually counted using Image J.

#### Black Gold II Myelin Stain

Following sectioning, coronal brain sections were directly mounted on glass object slides and subjected to staining using the Black Gold II (BGII) myelin staining kit (3 mg/ml, Sigma-Aldrich), following manufacturer's instructions. The stained sections were then visualized using an Olympus BH2 microscope equipped with a monochrome digital camera (Leica Microsystems). Images were obtained with Leica QWin Software. ImageJ software was used to measure the relative optical density (OD) in the corpus callosum (CC), up to two sections per animal were analysed.

#### Statistical Analysis

Data are presented as mean values with standard error of mean (SEM) unless otherwise stated. Normal distribution of the data was tested with the Shapiro–Wilk test. Statistical analyses were performed with the Kruskal–Wallis test followed by Dunn’s post hoc test when analysing percentages. Body weight and PET signal changes over time were tested with the repeated measures general linear model with the Sidak’s multiple comparison test to compare between time points. Differences in behavioural outputs and cell count were tested with one-way ANOVA followed by the Tukey’s post hoc test. A value of p < 0.05 was considered statistically significant. Statistics and data plotting were performed with GraphPad Prism Software v8 (San Diego, California). No animals were excluded from the behavioural tests and PET analysis. During the analysis of the immunohistochemical staining, some slices were damaged in the process or had to be excluded because of clear technical artifacts. Individual animals are also plotted as dots in the bar graphs. Statistical differences in the graphs are reported against the CPZ group at end of exposure to the CPZ-containing diet.

## Results

### Cuprizone Feeding Led to Motor Skill Impairments in the Balance Beam and MOSS Tests

**Fig. 2:**
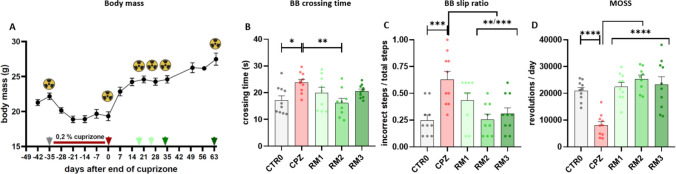
Cuprizone feeding leads to behavioural impairments in the balance beam and motor skill sequence (MOSS) tests. C57BL/6 male mice were fed with 0.2% cuprizone for five weeks and sacrificed before (CTR0, grey) or at the end of cuprizone feeding (CPZ, red) as well as 18 (RM1, green), 26 (RM2, green), 34 (RM3, green) and 63 days (RM4, green) after cuprizone feeding was stopped. Body mass was measured daily and plotted as weekly average (**A**). Before sacrifice, mice performed two behavioural tests: the balance beam (BB, **B** and **C**) and the MOSS (**D**) tests. The time taken to cross the beam was recorded (**B**) and the number of incorrect and total steps were counted and presented as ratio (**C**). The cages were provided with a wheel with irregular spaced crossbars and wheel revolutions per day were measured (**D**). n = 9–10/group, data are presented as mean ± SEM, differences in body mass over time were tested with repeated measures general linear model followed by Sidak post hoc test (A, F(14,1) = 104.020, p < 0.0001) while behavioural changes from CPZ group were tested with one-way ANOVA followed by Tukey’s post hoc test (C-D,*p < 0.05, **p < 0.01, ***p < 0.001, ****p < 0.0001)

The dietary CPZ model is frequently used to investigate the mechanisms and effects of de- and remyelination [9]. Here, animals were fed with 0,2% CPZ powder mixed in standard chow for five weeks, followed by normal diet (Fig. 1). Cuprizone feeding has a clear effect on growth and body mass of the mice. Figure 2A shows the body mass of the mice in the RM4 group (n = 9) that were longitudinally imaged and available at all time points. Mice lost around 15% of their weight during CPZ intoxication, but they also quickly started gaining body mass after CPZ feeding was terminated (Fig. 2A).

To assess the effects of CPZ on motor coordination, the time to cross a beam along with the number of missed steps was measured in the balance beam test. The crossing time in the CPZ group (23.98 ± 1.2 s, n = 10) was significantly longer than crossing time in the CTR0 (17.17 ± 1.7 s, n = 10) and RM2 groups (16.19 ± 1.7 s, n = 9; F_4,43_ = 3.945, *p* =  0.0082; Fig. 2B), not compared to the RM1 (n = 9) and RM3 (n = 10) groups. Furthermore, the slip ratio (incorrect steps/total steps) is considered to be a more sensitive measure in this test. It indeed showed a more gradual recovery pattern given that the slip ratio of the RM1 group (0.43 ± 0.07, n = 9) lies in between the CPZ (0.63 ± 0.07, n = 10) and the slip ratio of the RM2 (0.25 ± 0.05, n = 9) and the RM3 (0.31 ± 0.05, n = 10) groups. CPZ mice computed a significantly higher slip ratio than the CTR0 (0.25 ± 0.04, n = 10), RM2 and RM3 groups (F_4,43_ = 7.046, *p* =  0.0002; Fig. 2C), but not the RM1 group. In the RM2 and RM3 groups, the slip ratio was back to baseline level (CTR0 group).

As a secondary measure of motor skills, the MOSS test was performed. Mice were exposed to a wheel with irregular spaced crossbars which requires motor coordination in order to keep the wheel rotating [35]. Mice fed with CPZ showed a > 50% reduction in the number of wheel revolutions per day (8101 ± 1349 rev/day, n = 10), compared to CTR0 mice (20988 ± 1163 rev/day, n = 10; F_4,44_ = 14.34, *p* <  0.0001). In this test, motor performance returned to baseline level already 18 days after the end of CPZ feeding (RM1; 22491 ± 1592 rev/day, n = 10; Fig. 2D). Taken together, feeding the mice with a CPZ-containing diet led to motor coordination deficits as measured by the balance beam and MOSS tests. The behavioural impairments measured after the CPZ diet reversed to baseline once mice were fed standard chow.

### Cuprizone Feeding Induced robust Demyelination in both Corpus Callosum and Cortex

**Fig. 3:**
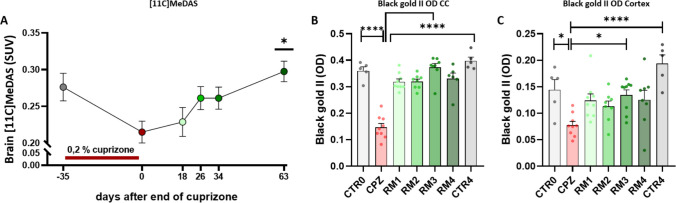
Remyelination following cuprizone-induced demyelination proceeds faster in the corpus callosum than in the cortex. C57BL/6 male mice were fed with 0.2% cuprizone for five weeks and sacrificed before (CTR0, grey) or at the end of cuprizone feeding (CPZ, red) as well as 18 (RM1, green), 26 (RM2, green), 34 (RM3, green) and 63 days (RM4, green) after cuprizone feeding was stopped or at 5.5 months old (CTR4, grey). To visualize myelin, PET imaging of [^11^C]MeDAS was performed longitudinally in the same animals (**A**, n = 8/group) and the standardized uptake value (SUV) in the brain was measured. Coronal brain sections were stained for Black Gold II (BGII) and the optical density of BGII signal was measured in the corpus callosum (**B**) and cortex (**C**). n = 5–9/group, data are presented as mean ± SEM, differences in [^11^C]MeDAS brain uptake over time (**A**) were tested with repeated measures general linear model followed by Sidak post hoc test (A, F(5,1) = 3.663, p = 0.009, *p < 0.05) while BGII optical density changes from CPZ group were tested with one-way ANOVA followed by Tukey’s post hoc test (**B-C**,*p < 0.05, ****p < 0.0001)

**Fig. 4:**
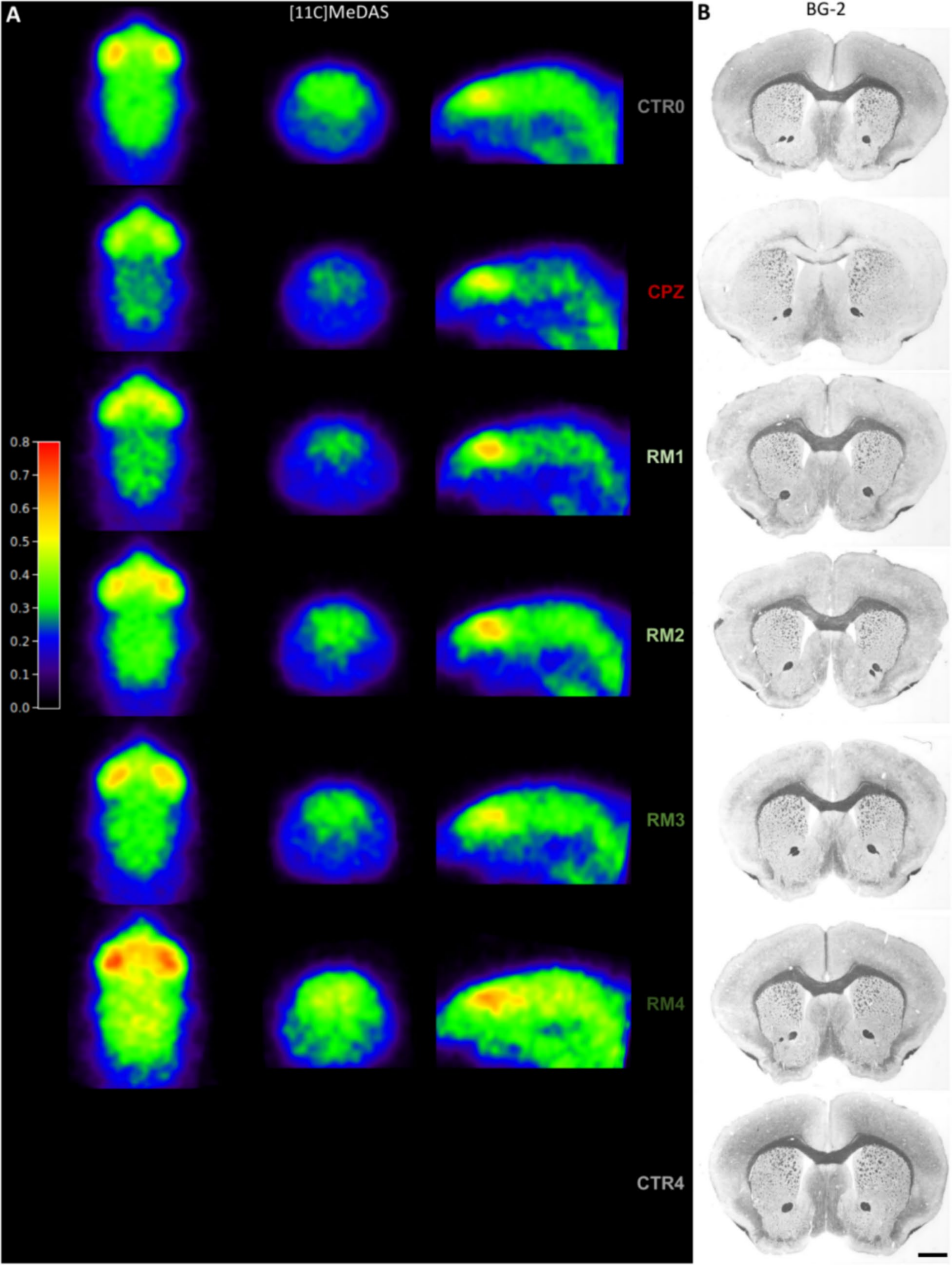
Average images (n = 8 at all included timepoints) for [^11^C]MeDAS brain SUV (**A**). From left to right: transversal, coronal and sagittal view. Corresponding representative coronal slices (Bregma = 0 to 1.10 mm) for BGII staining (**B**) at the same timepoints with CTR4 in addition (scale bar: 1 mm)

To visualize the extent of CPZ-induced demyelination, PET imaging was performed in the same animals (n = 8) over time using [^11^C]MeDAS as the tracer. The PET tracer [^11^C]MeDAS binds to β-sheet structures found in intact MBP was therefore used for relative quantification of myelin content. Whole brain uptake of [^11^C]MeDAS had decreased after CPZ feeding (0.22 ± 0.02 SUV), without reaching significance when compared to the CTR0 group (0.28 ± 0.02 SUV), and it steadily increased during the remyelination phase, reaching tracer uptake levels similar to baseline at RM2 (0.26 ± 0.02 SUV; Fig. 3A, 4A and Table S1). Interestingly, the [^11^C]MeDAS signal increased even more at 63 days (RM4; 0.30 ± 0.01) and was significantly different from the CPZ group (*p* =  0.01).

To confirm the effects of CPZ on myelination measured by PET imaging, mice were sacrificed at different time points, and brain sections were stained with Black Gold II. Mice terminated immediately after 5-weeks of CPZ diet showed lower levels of myelin in both CC (0.15 ± 0.01 OD, n = 9) and cortex (0.08 ± 0.01 OD, n = 9) than any other mice (Fig. 3B and C; Fig. 4B). Interestingly, myelin was restored faster in the CC (RM1: 0.32 ± 0.01 OD, n = 9; Fig. 3B) than in the cortex (RM1: 0.12 ± 0.01 OD, n = 9; Fig. 3C), the latter reaching similar levels to baseline only 34 days after the end of CPZ feeding (0.13 ± 0.01 OD, n = 9; RM3). In the CC, age-matched control mice (CTR4: 0.39 ± 0.01 OD, n = 5) sacrificed at the same time as RM4 show slightly higher myelin levels than RM4 (0.33 ± 0.02 OD, n = 6) and CTR0 mice (0.36 ± 0.01 OD, n = 5). Similarly, CTR4 mice (0.19 ± 0.02 OD, n = 5) also show higher myelin than RM4 (0.12 ± 0.02 OD, n = 8) and CTR0 mice (0.14 ± 0.02 OD, n = 5) in the cortex. These last reported effects are not significant but they suggest that myelin abundance might be affected by age. Collectively, CPZ-fed mice showed extensive myelin loss at the end of the CPZ-feeding period, as shown by PET imaging and histological brain analysis. The reversibility of this dietary model is confirmed by the increased myelin content measured at different time points of remyelination.

### Cuprizone Feeding Enhanced Glial Responses in the Corpus Callosum and Cortex

**Fig. 5:**
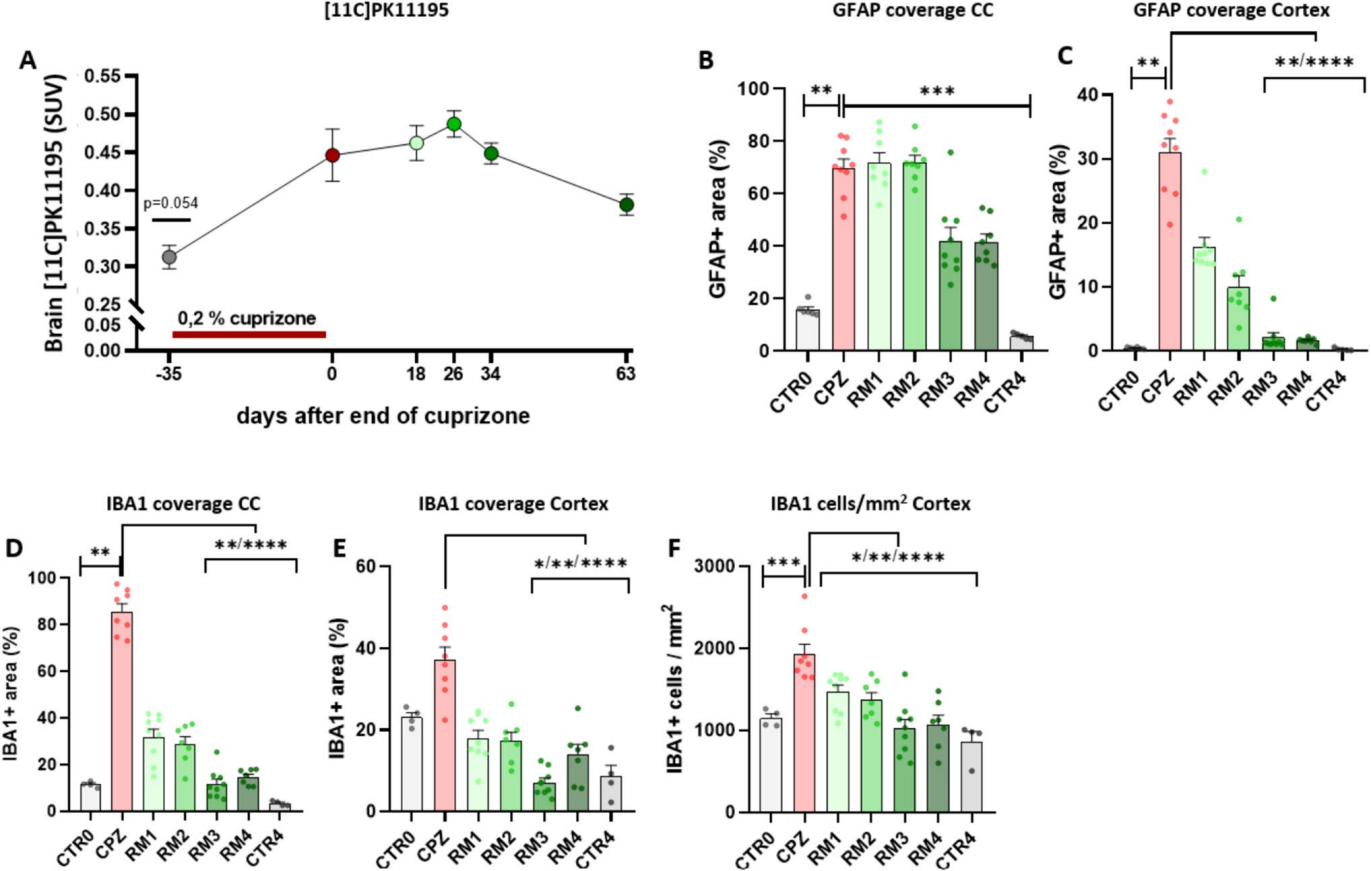
Cuprizone-induced demyelination enhances microglial and astrocytic responses in the corpus callosum and cortex. C57BL/6 male mice were fed with 0.2% cuprizone for five weeks and sacrificed before (CTR0, grey) or at the end of cuprizone feeding (CPZ, red) as well as 18 (RM1, green), 26 (RM2, green), 34 (RM3, green) and 63 days (RM4, green) after end of cuprizone feeding or at 5.5 months old (CTR4, grey). To visualize neuroinflammation, PET imaging of [^11^C]PK11195 was performed longitudinally in the same animals (**A**, n = 8/group) and the standardized uptake value (SUV) in the brain was measured. Coronal brain sections were stained for GFAP (**B** and **C**) or IBA1 (**D**-**F**) to visualize reactive astrocytes or microglia, respectively. Signal coverages as percentage area were quantified in the corpus callosum (**B** and **D**) and in the cortex (**C** and **E**). IBA1 positive cells were counted in cortical areas just above the corpus callosum (**F**). n = 5–9/group, data are presented as mean ± SEM, differences in [^11^C]PK11195 brain uptake over time were tested with repeated measures general linear model followed by Sidak post hoc test (A, F(5,1) = 11.931, p < 0.0001), changes in IBA1 or GFAP signal coverages from CPZ group were tested with Kruskal–Wallis test followed by Dunn’s post hoc test (**B-E**,*p < 0.05, **p < 0.01, ***p < 0.001,****p < 0.0001) and differences in IBA1 positive cells count from CPZ group were tested with one-way ANOVA (treatment effect: F(6,41) = 11.6, p < 0.0001) followed by Tukey’s post hoc test (**F**,*p < 0.05, **p < 0.01, ***p < 0.001,****p < 0.0001)

**Fig. 6:**
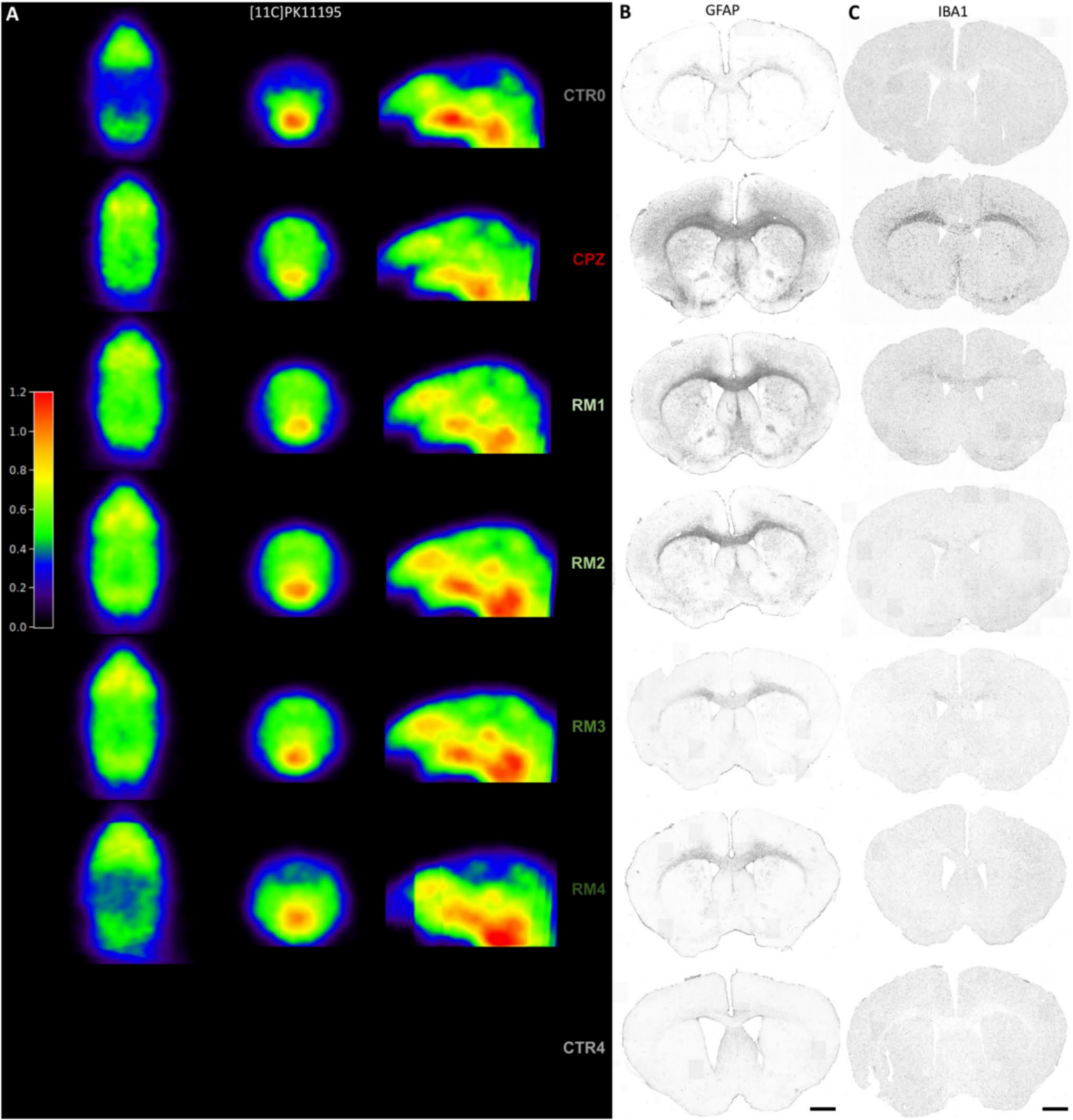
Average images (n = 8 at all included timepoints) for [^11^C]PK11195 brain SUV (**A**). From left to right: transversal, coronal and sagittal view. Corresponding representative coronal slices (Bregma = 0 to 1.10 mm) for GFAP staining (**B**) and IBA1 staining (**C**) at the same timepoints with CTR4 in addition (scale bar: 1 mm)

Neuroinflammation develops simultaneously to CPZ-induced demyelination. Reactive astrocytes and microglia respond quickly after the toxic insult, astrocytes being the faster responders recruiting microglia to clear myelin debris [40]. To measure the neuroinflammatory response over time following CPZ-induced demyelination, [^11^C]PK11195 PET was used [30] in the same animals (n = 8) over time. [^11^C]PK11195 binds to TSPO, a mitochondrial outer membrane receptor thought to be mainly overexpressed in the brain by reactive astrocytes and microglia [26]. A trend towards increased brain uptake of [^11^C]PK11195 (*p* =  0.054) was observed immediately after CPZ feeding (0.45 ± 0.03 SUV), when compared to the CTR0 group (0.312 ± 0.015 SUV). Tracer levels remained elevated until 34 days after the end of CPZ (RM3, 0.45 ± 0.01 SUV; Fig. 5A, 6A and Table S2). Even though a decrease in signal between days 34 and 63 could be appreciated, [^11^C]PK11195 uptake on day 63 (RM4 = 0.38 ± 0.01 SUV) remained elevated when compared to baseline.

To further assess the contribution of astrocytes and microglia to the neuroinflammatory response, brain sections were stained with GFAP and IBA1, respectively. Immediately after cessation of CPZ feeding, GFAP signal coverage was drastically increased in both CC (70 ± 3%, n = 9) and cortex (31 ± 2%, n = 9; Fig. 5B and C; Fig. 6B and C) when compared to the CTR0 group (CC: 16 ± 1%, n = 6; cortex: 0.43 ± 0.06%, n = 6; **p <  0.01). In the CC, GFAP signal persisted at elevated levels until RM3 (42 ± 5%, n = 9, **p <  0.01) and did not reach baseline at RM4 (42 ± 3%, n = 8, **p <  0.01; Fig. 5B). On the other hand, the GFAP signal in the cortex greatly decreased as soon as 18 days after the end of CPZ treatment (RM1, 16 ± 2%, n = 9) and reached control levels at RM4 (1.6 ± 0.14%; Fig. 5C, n = 8). Furthermore, coverage of IBA1 signal in both CC (86 ± 3%, n = 8) and cortex (37 ± 3%, n = 8) was higher immediately after CPZ-feeding than at any other time point (Fig. 5D and E). In the CC, IBA1 signal reached baseline levels at RM3 (12 ± 2%, n = 9) and RM4 (15 ± 1%, n = 8), but age-matched controls (CTR4) showed even lower signal coverage at day 63 (3.4 ± 0.5%, n = 4; Fig. 5D). In the cortex, IBA1 signal was generally lower than the signal in CC and the CPZ-induced increase in coverage is reversed as early as RM1 (Fig. 5E). CPZ feeding also significantly increased the number of IBA1 positive cells (1931 ± 120 cells/mm^2^, n = 8) when compared to CTR0 group (1150 ± 51 cells/mm^2^, n = 4, ***p <  0.001), which decreased gradually over time reaching baseline level at RM3 (1023 ± 110 cells/mm^2^, n = 9, ****p <  0.0001) and RM4 (1070 ± 113, n = 8, ****p <  0.0001; Fig. 5F). In general, CPZ feeding led to enhanced astrocytic and microglial responses which remained altered in the CC long after CPZ was removed from the diet, especially for astrocytes.

## Discussion

The present study investigated the behavioural and neuropathological changes in mice fed with CPZ for five weeks, followed by examination of long-term effects using a longitudinal approach. Our results provide new insights into the reversibility of CPZ-induced demyelination, neuroinflammation and the associated behavioural deficits, shedding light especially on the temporal dynamics of these processes. We found that feeding mice with CPZ for five weeks leads to transient myelin loss and motor impairments while inducing persistent glial activation, especially astrogliosis in the CC. We further elucidated the ability of [^11^C]MeDAS and [^11^C]PK11195 PET tracers to track long-term changes in CPZ-induced myelination and neuroinflammation, respectively.

Consistent with previous findings [32, 35, 41], our study demonstrated that CPZ feeding led to motor skill impairments, as evident in the increase in crossing time and the number of missed steps in the balance beam test. The level of these impairments was in agreement with the reduced voluntary running wheel activity observed in the MOSS test, which also reflects compromised motor coordination during CPZ intoxication. Importantly, our longitudinal analysis revealed that these behavioural deficits were reversible, with improvements in both balance beam and MOSS tests during the remyelination phase. This indicated that the observed motor coordination deficits are directly linked to the CPZ-induced demyelination and support the validity of these behavioural paradigms for studying the effects of demyelination. Given the more gradual decrease in missteps compared to the rapid increase in wheel revolutions, the balance beam test, especially the slip ratio parameter, was more sensitive in measuring motor impairments during the remyelination phase than the MOSS test. After CPZ-induced demyelination, however, the MOSS test seemed to be a sensitive test for myelin repair in CC, as the performance in this test quickly returned to baseline level, coinciding with the fast recovery of remyelination in the CC in the Black Gold II staining. These coinciding effects could explain a restored hemispheric communication underlying paw coordination. In contrast, both beam crossing time and missed steps took longer to ameliorate, as was also observed for remyelination in the cortex. Based on these findings, both behavioural tests can assess cuprizone-induced motor skills impairments, reflecting different aspects of the remyelination process: the MOSS test seems to mirror myelin repair in the CC whereas the balance beam test seems to better assess remyelination in the cortex.

PET imaging with [^11^C]MeDAS provided a quantitative assessment of myelin content in the brains of CPZ-fed mice. The average brain uptake values for this tracer showed a clear effect over time, but post-hoc significant differences could only be detected between the CPZ and RM4 groups, because of high variability in the measurements. Future studies employing this tracer should probably be performed with more animals to increase statistical power. The decrease in brain uptake of [^11^C]MeDAS during cuprizone feeding and subsequent recovery during remyelination phases corroborated the de- and remyelination dynamics observed in the histological analysis. However, the [^11^C]MeDAS brain uptake in the RM1 group remains at a similar level to CPZ group while BGII staining at RM1 is comparable to baseline (CTR0). This is in line with previous findings [25] and [^11^C]MeDAS may bind only to complete mature myelin, thereby underestimating the actual myelin content present. Additionally, [^11^C]MeDAS brain SUV is higher at RM4 than at baseline (CTR0) and this difference cannot be observed in the BGII staining. Nevertheless, BGII staining also showed a stronger signal in age-matched controls (CTR4) than CTR0 mice. This may suggest an effect of age on myelin content. The brain metabolome atlas shows changes in myelinating processes from adolescence to adulthood that may strengthen the stability of myelin structures [42]. In line with a previous study [43], [^11^C]MeDAS performed well in detecting changes in overall myelin content in the CPZ model, but the technique is not suitable for differentiating the effect in different brain regions in mice, due to the limited spatial resolution of the PET camera and the small size of the mouse brain. Similarly, lesions in the EAE rat model were not detected by [^11^C]MeDAS, which was also ascribed to the low resolution of the PET camera to detect small-sized lesions [29]. The discrepancies between PET and IHC shown here and elsewhere [20] underscore the need for new PET tracers [44] and higher resolution PET cameras able to accurately track the kinetics of de- and remyelination processes to further guide the development of novel therapeutics enhancing remyelination.

As a hallmark of demyelinating diseases, neuroinflammation was assessed using the [^11^C]PK11195 PET tracer targeting TSPO. The sustained elevation of [^11^C]PK11195 brain uptake even at later time points after the cessation of CPZ feeding suggests a prolonged neuroinflammatory response. This was further supported by IHC analysis showing increased GFAP and IBA1 signals immediately after cessation of CPZ feeding, indicative of reactive astrocytes and microglia activation, respectively. Notably, the astrocytic response in the CC remained elevated even at RM4, suggesting a persistent astrogliosis in this region, while the cortex showed a more rapid recovery. This is comparable with previous findings showing elevated area coverage by astrocytes after six weeks of CPZ-feeding [45]. Moreover, persistent astrocyte activation has been reported up to seven months after a five-week CPZ diet, despite normal myelin density [17]. In that study, as well as in another investigation using a comparable experimental design [18], evidence of underlying axonal degeneration was also observed. Together, these findings raise the possibility that slowly progressive axonal degeneration may occur during the remyelination phase, accompanied by sustained astrocytic activation. When comparing PET and IHC data, changes in PET signal seem to reflect more astrocyte behaviour than microglia, as the change in GFAP signal in CC changed over time in a similar fashion as the PET signal, whereas the IBA1 signal decreased more rapidly over time. Previous literature shows that microglia are mainly expressing TSPO after four weeks of CPZ feeding, while astrocytes primarily contribute after six weeks [46]. Notably, TSPO expression in microglia is differentially regulated between mice and humans. While in rodent models of neurodegenerative diseases TSPO can be used as a marker for activated microglia, in human *postmortem* samples TSPO expression was not changed [47]. Furthermore, TSPO expression level reflects density of microglia rather than their activation state in MS lesions [48]. These findings emphasize that the cellular source of TSPO reflected in the PET signal and its translational value should be interpreted carefully.

In conclusion, our study contributed to the growing body of knowledge on the CPZ model, emphasizing its utility for studying de- and remyelination processes and associated behavioural changes. The findings show that CPZ diet induces transient demyelination and motor deficits which recover quickly after cuprizone withdrawal. Nevertheless, astrocyte activation persists for a long period of time, especially in the CC. Further, [^11^C]MeDAS tracer may not be suited to track early remyelination processes in detail, especially at specific lesion sites and [^11^C]PK11195 signal may represent astrocytic rather than microglia responses. This study offers a comprehensive and longitudinal analysis of the effects up to two months after a five-week CPZ diet. It provides valuable insights for future research using PET imaging in the context of demyelinating diseases, especially for long-term therapeutic interventions.

## Supplementary Information

Below is the link to the electronic supplementary material.

Supplementary file1 (DOCX 30 KB)

## Data Availability

All research data and computer codes are available from the corresponding author upon request.
