# Supplementary material for: Astrocyte Activation Persists after Recovery of Myelin and Motor Deficits in the Cuprizone Model: a Longitudinal PET and CNS Tissue Analysis Study
Source: Mol Imaging Biol. 2026 Mar 24;28(3):457–69. doi: 10.1007/s11307-026-02098-5 (PMC13337915; doi:10.1007/s11307-026-02098-5)
Supplement: Supplementary file 1 — Supplementary file1 (DOCX 30 KB) [file 11307_2026_2098_MOESM1_ESM.docx]

| **[^11^C]MeDAS** | | | | | | | | | | | | | |
| --- | --- | --- | --- | --- | --- | --- | --- | --- | --- | --- | --- | --- | --- |
|  |  | **CTR0** | | **CPZ** | | **RM1** | | **RM2** | | **RM3** | | **RM4** | |
| **brain region** | **volume (mm^3^)** | avg | *std* | avg | *std* | avg | *std* | avg | *std* | avg | std | avg | *std* |
|  |  |  |  |  |  |  |  |  |  |  |  |  |  |
| amygdala | 10,10 | 0,33 | *0,06* | 0,26 | *0,06* | 0,27 | *0,07* | 0,32 | *0,05* | 0,31 | *0,04* | 0,35 | *0,04* |
| anterior commissure | 1,06 | 0,35 | *0,06* | 0,29 | *0,08* | 0,31 | *0,08* | 0,35 | *0,05* | 0,34 | *0,05* | 0,40 | *0,06* |
| basal forebrain & septum | 15,26 | 0,34 | *0,06* | 0,27 | *0,06* | 0,29 | *0,07* | 0,33 | *0,05* | 0,32 | *0,05* | 0,38 | *0,05* |
| caudate putamen | 24,89 | 0,33 | *0,06* | 0,25 | *0,06* | 0,27 | *0,07* | 0,30 | *0,05* | 0,30 | *0,05* | 0,35 | *0,05* |
| central gray | 3,90 | 0,31 | *0,07* | 0,25 | *0,05* | 0,25 | *0,06* | 0,30 | *0,07* | 0,28 | *0,04* | 0,35 | *0,07* |
| cerebellum | 58,55 | 0,24 | *0,05* | 0,18 | *0,03* | 0,21 | *0,04* | 0,24 | *0,05* | 0,23 | *0,04* | 0,26 | *0,03* |
| corpus callosum | 15,37 | 0,27 | *0,06* | 0,20 | *0,05* | 0,21 | *0,05* | 0,24 | *0,04* | 0,25 | *0,05* | 0,28 | *0,04* |
| fimbria | 1,50 | 0,32 | *0,07* | 0,24 | *0,05* | 0,25 | *0,06* | 0,29 | *0,05* | 0,29 | *0,05* | 0,34 | *0,05* |
| globus pallidus | 2,22 | 0,35 | *0,06* | 0,27 | *0,06* | 0,30 | *0,09* | 0,33 | *0,06* | 0,32 | *0,04* | 0,36 | *0,05* |
| hippocampus | 28,41 | 0,30 | *0,06* | 0,23 | *0,05* | 0,24 | *0,06* | 0,28 | *0,05* | 0,27 | *0,05* | 0,32 | *0,04* |
| hypothalamus | 12,71 | 0,34 | *0,06* | 0,29 | *0,07* | 0,29 | *0,07* | 0,33 | *0,05* | 0,33 | *0,04* | 0,37 | *0,04* |
| inferior colliculus | 6,18 | 0,24 | *0,05* | 0,19 | *0,04* | 0,21 | *0,04* | 0,23 | *0,06* | 0,21 | *0,05* | 0,25 | *0,05* |
| internal capsule | 2,53 | 0,35 | *0,05* | 0,26 | *0,06* | 0,29 | *0,08* | 0,32 | *0,06* | 0,31 | *0,04* | 0,36 | *0,05* |
| neocortex | 141,06 | 0,24 | *0,05* | 0,19 | *0,04* | 0,20 | *0,05* | 0,22 | *0,04* | 0,24 | *0,04* | 0,26 | *0,04* |
| rest of midbrain | 13,90 | 0,34 | *0,06* | 0,27 | *0,06* | 0,29 | *0,07* | 0,33 | *0,05* | 0,32 | *0,05* | 0,38 | *0,05* |
| superior colliculus | 10,42 | 0,28 | *0,06* | 0,21 | *0,04* | 0,21 | *0,04* | 0,24 | *0,06* | 0,23 | *0,05* | 0,28 | *0,05* |
| thalamus | 27,77 | 0,33 | *0,06* | 0,27 | *0,06* | 0,29 | *0,08* | 0,32 | *0,05* | 0,31 | *0,05* | 0,36 | *0,05* |
| ventricles | 8,01 | 0,32 | *0,06* | 0,24 | *0,06* | 0,26 | *0,06* | 0,30 | *0,05* | 0,30 | *0,05* | 0,34 | *0,04* |
|  |  |  |  |  |  |  |  |  |  |  |  |  |  |
| brainstem | 80,35 | 0,30 | *0,05* | 0,25 | *0,05* | 0,27 | *0,07* | 0,32 | *0,05* | 0,30 | *0,04* | 0,33 | *0,03* |
| olfactory bulb | 29,25 | 0,33 | *0,05* | 0,28 | *0,05* | 0,30 | *0,07* | 0,33 | *0,05* | 0,34 | *0,04* | 0,36 | *0,04* |

**Table S1** Mouse brain region analysis of [^11^C]MeDAS PET imaging.

Avg: average (n=8 mice); CTR: control ; CPZ: cuprizone; RM1-4: remyelination timepoints; std: standard deviation

**Table S2** Mouse brain region analysis of [^11^C]PK11195 PET imaging.

| **[^11^C]PK11195** | | | | | | | | | | | | | |
| --- | --- | --- | --- | --- | --- | --- | --- | --- | --- | --- | --- | --- | --- |
|  |  | **CTR0** | | **CPZ** | | **RM1** | | **RM2** | | **RM3** | | **RM4** | |
| **brain region** | **volume (mm^3^)** | avg | *std* | avg | *std* | avg | *std* | avg | *std* | avg | *std* | avg | *std* |
|  |  |  |  |  |  |  |  |  |  |  |  |  |  |
| Amygdala | 10,10 | 0,42 | *0,07* | 0,52 | *0,12* | 0,54 | *0,06* | 0,57 | *0,04* | 0,54 | *0,03* | 0,50 | *0,08* |
| anterior commissure | 1,06 | 0,42 | *0,07* | 0,58 | *0,13* | 0,58 | *0,08* | 0,60 | *0,03* | 0,57 | *0,05* | 0,51 | *0,02* |
| basal forebrain & septum | 15,26 | 0,42 | *0,06* | 0,57 | *0,12* | 0,58 | *0,08* | 0,60 | *0,04* | 0,57 | *0,04* | 0,50 | *0,05* |
| caudate putamen | 24,89 | 0,31 | *0,04* | 0,55 | *0,12* | 0,53 | *0,08* | 0,56 | *0,06* | 0,51 | *0,04* | 0,40 | *0,04* |
| central gray | 3,90 | 0,31 | *0,05* | 0,43 | *0,10* | 0,47 | *0,07* | 0,49 | *0,04* | 0,46 | *0,03* | 0,31 | *0,07* |
| cerebellum | 58,55 | 0,35 | *0,04* | 0,38 | *0,09* | 0,45 | *0,07* | 0,50 | *0,06* | 0,46 | *0,05* | 0,39 | *0,04* |
| corpus callosum | 15,37 | 0,25 | *0,03* | 0,46 | *0,10* | 0,46 | *0,07* | 0,47 | *0,06* | 0,43 | *0,05* | 0,34 | *0,04* |
| Fimbria | 1,50 | 0,30 | *0,04* | 0,56 | *0,13* | 0,56 | *0,08* | 0,59 | *0,04* | 0,55 | *0,04* | 0,40 | *0,06* |
| globus pallidus | 2,22 | 0,33 | *0,04* | 0,55 | *0,11* | 0,54 | *0,09* | 0,58 | *0,08* | 0,52 | *0,03* | 0,41 | *0,07* |
| hippocampus | 28,41 | 0,31 | *0,04* | 0,47 | *0,10* | 0,48 | *0,07* | 0,51 | *0,04* | 0,47 | *0,04* | 0,37 | *0,07* |
| hypothalamus | 12,71 | 0,48 | *0,08* | 0,59 | *0,14* | 0,61 | *0,07* | 0,63 | *0,06* | 0,57 | *0,06* | 0,55 | *0,06* |
| inferior colliculus | 6,18 | 0,34 | *0,04* | 0,53 | *0,12* | 0,55 | *0,08* | 0,57 | *0,04* | 0,54 | *0,03* | 0,42 | *0,05* |
| internal capsule | 2,53 | 0,30 | *0,04* | 0,53 | *0,12* | 0,54 | *0,08* | 0,57 | *0,06* | 0,52 | *0,04* | 0,40 | *0,08* |
| Neocortex | 141,06 | 0,27 | *0,04* | 0,40 | *0,09* | 0,40 | *0,06* | 0,42 | *0,05* | 0,38 | *0,04* | 0,35 | *0,03* |
| rest of midbrain | 13,90 | 0,42 | *0,06* | 0,57 | *0,12* | 0,58 | *0,08* | 0,60 | *0,04* | 0,57 | *0,04* | 0,50 | *0,05* |
| superior colliculus | 10,42 | 0,29 | *0,04* | 0,40 | *0,08* | 0,44 | *0,07* | 0,47 | *0,06* | 0,42 | *0,04* | 0,31 | *0,07* |
| Thalamus | 27,77 | 0,31 | *0,04* | 0,51 | *0,11* | 0,51 | *0,09* | 0,55 | *0,04* | 0,51 | *0,02* | 0,38 | *0,06* |
| ventricles | 8,01 | 0,34 | *0,04* | 0,53 | *0,12* | 0,55 | *0,08* | 0,57 | *0,04* | 0,54 | *0,03* | 0,42 | *0,05* |
|  |  |  |  |  |  |  |  |  |  |  |  |  |  |
| brainstem | 80,35 | 0,43 | *0,06* | 0,47 | *0,11* | 0,50 | *0,07* | 0,54 | *0,02* | 0,55 | *0,03* | 0,49 | *0,08* |
| olfactory bulb | 29,25 | 0,46 | *0,08* | 0,53 | *0,11* | 0,54 | *0,09* | 0,56 | *0,03* | 0,55 | *0,03* | 0,53 | *0,12* |

Avg: average (n=8 mice); CTR: control ; CPZ: cuprizone; RM1-4: remyelination timepoints; std: standard deviation
